# Biological characteristics of transcription factor RelB in different immune cell types: implications for the treatment of multiple sclerosis

**DOI:** 10.1186/s13041-019-0532-6

**Published:** 2019-12-27

**Authors:** Meng-ge Yang, Li Sun, Jinming Han, Chao Zheng, Hudong Liang, Jie Zhu, Tao Jin

**Affiliations:** 1 Department of Neurology and Neuroscience Center, The First Hospital of Jilin University, Xinmin Street 71#, Changchun, 130021 China; 2 Present address: Department of Clinical Neuroscience, Karolinska Institute, Stockholm, Sweden; 3 Department of Neurobiology, Care Sciences and Society, Karolinska Institute, Karolinska University Hospital Huddinge, Stockholm, Sweden

**Keywords:** RelB, NF-κB, Multiple sclerosis, Experimental autoimmune encephalomyelitis, Neuroinflammation

## Abstract

Transcription factor RelB is a member of the nuclear factror-kappa B (NF-κB) family, which plays a crucial role in mediating immune responses. Plenty of studies have demonstrated that RelB actively contributes to lymphoid organ development, dendritic cells maturation and function and T cells differentiation, as well as B cell development and survival. RelB deficiency may cause a variety of immunological disorders in both mice and humans. Multiple sclerosis (MS) is an inflammatory and demyelinating disease of the central nervous system which involves a board of immune cell populations. Thereby, RelB may exert an impact on MS by modulating the functions of dendritic cells and the differentiation of T cells and B cells. Despite intensive research, the role of RelB in MS and its animal model, experimental autoimmune encephalomyelitis, is still unclear. Herein, we give an overview of the biological characters of RelB, summarize the updated knowledge regarding the role of RelB in different cell types that contribute to MS pathogenesis and discuss the potential RelB-targeted therapeutic implications for MS.

## Introduction

Transcription factors of the nuclear factor-kappa B (NF-κB) family play a critical role in regulating the innate and adaptive immune responses [1]. In mammalian cells, this family includes five members: c-Rel, p65 (RelA), RelB, p105/p50 (NF-κB1) and p100/p52 (NF-κB2) [2]. RelB, first described in 1992 [3], is considered an extraordinary member of the NF-κB family with diverse and unique features [4–7]. Over the past two decades, a variety of biological characteristics and immunological effects of RelB have been reported [8, 9].

Multiple sclerosis (MS) is a neuro-inflammatory disease that is mainly characterized by multicentric white matter demyelination of the central nervous system (CNS) [10]. It is a disabling disease that primarily affects young adults, particularly young women [11]. MS can be commonly clinically classified into four categories, including relapsing-remitting MS (RRMS), primary progressive MS (PPMS), secondary progressive MS (SPMS) and progressive relapsing MS (PRMS), among which RRMS is the most common and classical form [12]. In the early course of MS, acute attacks of neurological impairment are followed by partial or complete remission. The relapsing-remitting neurological dysfunctions lead to chronic neurological damage and neurodegeneration, which results in disability accumulation and disease progression. The clinical symptoms of MS are complex and diverse, including motor, sensory, visual and autonomic system dysfunctions [13, 14]. Current disease-modifying therapies could reduce the frequency of relapses; however, the progression of MS can be not effectively prevented [15–17]. Hence, new therapeutic strategies for MS still need to be proposed.

Given the significant role of NF-κB in immune response [18–21], a better understanding of the role of RelB in MS is potentially beneficial for exploring the pathogenesis and looking for new immunotherapies for treatment. This review will outline the features of RelB and RelB-associated pathogenic mechanisms in MS, as well as the therapeutic implications of targeting RelB.

## Biological characteristics of transcription factor RelB

### The gene structure and expression of RelB

The latest data has illustrated that the human RelB gene is located on chromosome 19q13.32, where 12 exons encode a protein with 579 amino acids [22]. The 5′ genomic region of RelB is characterized by a TATA-less promoter containing two κB cis-acting sites. Furthermore, potential vitamin D response elements have been recognized in the RelB promoter region, which are essential for negative transcriptional regulation and mediated by 1,25-Dihydroxyvitamin D_3_ (1,25-(OH)_2_D_3_) and its analogs [23]. Higher expression of the RelB gene is observed in the thymus medulla, the periarterial lymphatic sheaths of the spleen and the deep cortex of the lymph nodes [6, 24]. At the cellular level, RelB expression is mainly restricted to dendritic cells (DCs) [24]. Furthermore, RelB can also be expressed in other immune cells, such as T cells, B cells and monocytes [6, 25–27].

### Protein structure and functions of RelB

The RelB protein contains three important domains: the C-terminal transcriptional activation domain (TAD), the Rel homology region (RHD) and the N-terminal leucine zipper domain (LZ) [28]. The RHD, highly conserved sequences on all NF-κB family members, consists of 300 amino acids and is responsible for dimerization, nuclear translocation and DNA-binding activity [29]. The TAD is indispensable, but not sufficient to motivate expression of NF-κB-dependent genes [7]. The LZ, recognized for its unique characteristics, differs from other family members and participates in activating transcription of target genes. The structural integrity of both N- and C-terminals domains is necessary for the fully transcriptional activity of RelB [7].

Nevertheless, RelB protein is unstable. In the cytoplasm of unstimulated cells, RelB prevents its degradation by forming a steady heterodimer with p100/p52 or p105/p50 [30]. Differing from the other NF-κB members, a stable RelB homodimer is nonexistent [31]. Accumulating evidence suggests that RelB can act as both an activator and a repressor to regulate NF-κB-responsive gene expression [3, 5]. In addition, RelB plays a dual regulatory role in targeting gene expression by recruiting co-activators or co-repressors, like human epithelial growth factor receptor 2 (HER2) [32], histone H3 lysine methyltransferase G9a [33] and death domain-associated protein (Daxx) [34]. The accurate mechanisms underlying these divergent functions are currently unclear. One widely accepted notion is that RelB post-translational modifications, such as phosphorylation [35–37], ubiquitination [38] and SUMOylation [39], have a direct effect on RelB transcriptional activity, which results in functional diversity [40].

### The RelB-associated activation pathways

**Fig. 1:**
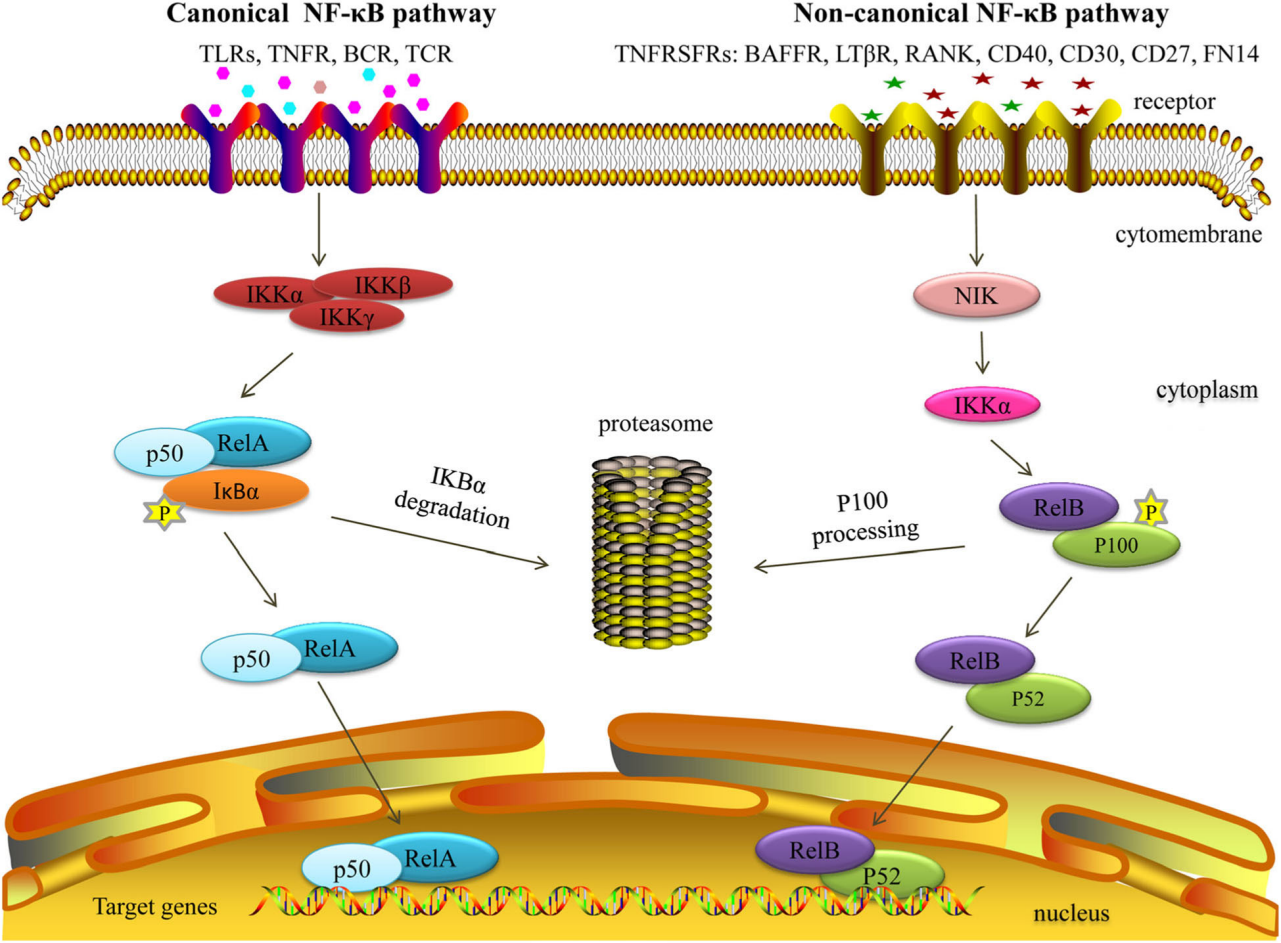
Canonical and non-canonical NF-κB pathways. The canonical pathway is triggered by various immune receptors, for example, TLRs, TNFR, BCR and TCR. Various receptors activate the IKK complex, resulting in phosphorylation and proteasome-dependent degradation of IκBα, which in turn frees RelA/p50 and promotes its nuclear import. The non-canonical pathway is induced by the TNFSFRs, such as BAFFR, LTβR, CD40 and RANK. Then, downstream molecules NIK and IKKα are activated, leading to p100 processing and the liberation of RelB/p52 heterodimers. Finally, the uncontrolled dimers translocate into nucleus and bind to target genes, triggering their expression Abbreviations: TLRs: Toll-like receptors; TNFR: tumor necrosis factor receptor; TCR: T cell receptor; BCR: B cell receptor; TNFSFRs: tumor necrosis factor superfamily receptors; BAFFR: B cell activating factor receptor; LTβR: lymphotoxin β receptor; RANK: receptor activator of NF-κB; FN14: fibroblast growth factor-inducible factor 14; IκB: κB inhibitor; IKK: IκB kinase; NIK: NF-κB-inducing kinase

NF-κB family members can be activated by either canonical or non-canonical NF-κB pathways. The canonical pathway can be triggered by various stimuli that bind to immune receptors, like the Toll-like receptors (TLRs), tumor necrosis factor receptor (TNFR), T cell receptor (TCR) and B cell receptor (BCR). Then, the inhibitor of κB kinase (IKK) complex, including two catalytic subunits IKKα and IKKβ, and one regulator IKKγ, can be activated, and in turn, phosphorylates IκBα (a member of κB inhibitors). After that, phosphorylated IκBα undergoes proteasome-dependent degradation and then releases the RelA/p50 complex. The freed RelA/p50 complex translocates into the nucleus and induces the expression of multiple inflammatory genes [41] (Fig. 1). The non-canonical NF-κB pathway is triggered by a series of tumor necrosis factor superfamily receptors (TNFSFRs) members, such as B cell activating factor receptor (BAFFR), lymphotoxin β receptor (LTβR), receptor activator of NF-κB (RANK), CD40, CD30, CD27 and fibroblast growth factor-inducible factor 14 (FN14) [42]. Once TNF superfamily molecules link to their corresponding TNFSFRs, NF-κB inducing kinase (NIK) phosphorylates and activates IKKα. The activated IKKα phosphorylates p100 at the site of C-terminal serine residues, leading to the partial degradation of p100 in the proteasome. Processing of p100 transforms it into p52, which then forms a RelB/p52 heterodimer that in turn migrates from the cytoplasm to the nucleus and promotes the expression of target genes through binding to the promoter or enhancer of target genes (Fig. 1) [43]. While the activation of the canonical pathway depends on the rapid and transient nuclear translocation of RelA/p50 dimers, the non-canonical pathway is activated in a slow and persistent manner via a RelB/p52 complex [2, 42]. Interestingly, the canonical and non-canonical pathways are not completely independent in most cases, but have an impact on each other [44]. The RelB-associated non-canonical pathway plays a critical role in regulating immune homeostasis, and its dysregulation contributes to inflammatory and autoimmune diseases [42, 43, 45–47].

### Immunomodulatory role of RelB

Accumulating evidence suggests that RelB deficiency can lead to a range of immune disorders in both mice and humans (Table 1) [9, 48]. In the following parts, we will discuss the role of RelB in immune organs, immune cells and immune responses.

**Table 1:** RelB deficiency can lead to a range of immune disorders in both mice and humans

Tissue or cell types	Immune disorders in mice with RelB deficiency	Immune disorders in humans with RelB deficiency	Related signaling	References
Thymus	highly disorganized medullary architecture; absent of mTECs and DCs, particularly Aire+ mTECs; impaired negative selection in T cells;	thymic dysplasia; lack of Hassall’s corpuscles;	LTβR CD40 RANK	[48–54]
SLOs	lack of Peyer’s patches and peripheric lymph nodes; splenic structural damage: impaired FDCs network; dispersed reticular fibroblast network throughout the white pulp; deficient GC and marginal zone development; decreased BCL and SCL production;	/	LTβR	[53, 55–57]
DCs	decreased surface markers: MHC class-II, CD11c, CD80, CD86 and CD40; lower capacity of antigen presentation and T cell activation;	/	AhR	[24, 52, 58–61]
T cells	reduced IFN-γ; damaged T cell differentiation and T cell immunity; multiorgan inflammation; significantly elevated migratory activity of effector memory T cell;	T cell dysmaturity; decreased T cell output from thymus; abnormal T cell subtypes clonal expansion; significantly reduced IFN-γ and IL-2 generation; decreased expression of T-bet and STAT1; evere T cell immunodeficiency;	/	[48, 53, 62–64]
B cells	reduced follicular B cells; Absent marginal zone B cells; B-cell progenitors developmental disorders; remarkable reduction of peripheral mature B cells;	barricaded B cell development; shortage of specific antibodies; severe B cell immunodeficiency; lack of CD27+ memory B cells; decreased expression of BAFFR; impaired CD40 signaling;	BAFFR CD40	[48, 56, 65–67]

Abbreviations: *mTECs* medullary thymic epithelial cells; *DCs* dendritic cells; *Aire* autoimmune regulator; *SLOs* secondary lymphoid organs; *FDCs* follicular dendritic cells; *GC* germinal center; *nTregs* natural regulatory T cells; *SLC* secondary lymphoid tissue chemokine; *BLC* B lymphocyte chemoattractant; *FoxP3* Forkhead box protein 3; *AhR* aryl hydrocarbon receptor; *IFN-γ* interferon-γ; *STAT1* signal transducer and activator of transcription 1; *RANK* receptor activator of NF-κB; *LTβR* lymphotoxin β receptor; *BAFFR* B cell activating factor receptor

#### Lymphoid organ development

Serving as the primary lymphoid organ, the thymus is a location for the development of T lymphocytes and the formation of central immunologic tolerance [68]. Thymus stromal cell microenvironments, in particular medullary thymic epithelial cells (mTECs), play a key role in these processes [69]. The mTECs are not only involved in the generation of Forkhead box protein 3-expressing regulatory T cells (FoxP3+ Tregs) [70], but can also express autoimmune regulator (Aire; Aire+ mTECs) that can contribute to negative thymocyte selection and suppress the initiation of autoimmune diseases [71–73]. The development of mTECs can be regulated by members of the TNFR superfamily, such as LTβR, CD40 and RANK, all of which can play their role through the canonical and non-canonical NF-κB pathways [74, 75]. Interestingly, a recent study revealed that the canonical pathways mediate mTECs differentiation by directly inducing RelB expression [49]. Acting mainly as a downstream signaling molecule of the TNFR superfamily, RelB is closely related to the development and functions of mTECs [50]. In RelB-deficient mice, the thymic medullary architecture is highly disorganized, mTECs and dendritic cells (DCs) are absent, and negative selection is impaired [49, 51–54]. Along this line, RelB deficiency in humans causes thymic dysplasia and decreased Hassall’s corpuscles [48]. Significantly, RelB is a necessary regulator for the expression of thymic Aire [54], and the development of Aire+ mTECs is primarily mediated by RANK signaling [76–79].

As secondary lymphoid organs (SLOs), the spleen, lymph nodes and Peyer’s patches provide accommodation for inactivated lymphocytes that can efficiently respond to diverse antigens, thereby making them essential for adaptive immunity [80]. An analysis of RelB-deficient mice suggested that RelB plays an important role in the development of secondary lymphoid organs. RelB-deficient mice lack Peyer’s patches and peripheral lymph nodes [53, 55]. Furthermore, RelB-deficient mice and spleens with severe structural damage, containing impaired follicular dendritic cells (FDCs) networks, a dispersed reticular fibroblast network throughout the white pulp, deficient germinal center (GC) and marginal zone development [56]. The anatomical imperfection in SLOs is closely related to the activation of the non-canonical NF-κB pathway by LTβR signaling via the RelB-related heterodimer [55–57, 81]. Once lymphotoxin-α_1_β_2_ (LTα_1_β_2_) expressed by lymphoid-tissue inducer cells binds to its relative LTβR, which is expressed by stromal organizer cells, non-canonical signaling is activated, inducing the expression of RelB-dependent homeostatic chemokines and cell adhesion molecules, which in turn attract and recruit lymphocytes to developing and mature SLOs [82]. During the expression of these homeostatic chemokines, secondary lymphoid tissue chemokine (SLC) and Epstein-Barr virus-induced molecule 1 ligand chemokine (ELC) are primarily responsible for the migration of T cells into SLOs, while B lymphocyte chemoattractant (BLC) plays a central role in attracting B cells [83, 84]. Furthermore, BCL and SCL generation can be prominently decreased in RelB-deficient mice [56]. Collectively, RelB is required by SLO formation and maintenance.

#### The maturation and function of DCs

DCs are professional antigen presenting cells (APCs), that are required for initiating adaptive immunity, since they provide signaling to antigen-specific naïve T cells that differentiate into functional mature T cells [85]. RelB plays a key role in DC maturation [24, 52, 58], particularly in myeloid-related DCs [86] that serve as conventional DCs (cDC) [87]. Surface markers associated with myeloid-derived DC maturation, such as major histocompatibility complex (MHC) class-II, CD11c, CD80, CD86 and CD40, were decreased in RelB-deficient mice. Furthermore, these deficiencies were not found in RelB-Venus knock-in mice [58]. RelB deficiency profoundly impaired DCs, both in their maturation and function [59]. In RelB-deficient bone marrow chimera mice, DCs showed a lower capacity of antigen presentation and T cell activation [59]. Aryl hydrocarbon receptor (AhR) signaling promotes RelB expression during DC maturation, and AhR deficiency in DCs may alter the control of RelB in DC maturation and function [60]. However, there are still different opinions on this topic. In 2017, Briseno et al. claimed that the development of most mouse cDC subsets did not rely on cell-intrinsic requirements for RelB [61]. Similarly, another study suggested that RelB/p50 promotes chemokine CCL19 expression instead of facilitating human DC maturation [88]. In summary, RelB plays key roles in the maturation and function of DCs. However, future studies are still needed to thoroughly investigate the association between RelB activation and DC development, making the present paradox clear.

#### T cell differentiation and T cell-mediated immunity

Responding to diverse antigens, T cells are involved in multiple processes of adaptive immunity [89]. During an immune response, activated naïve T cells can differentiate into effector cells and memory T cells in order to eliminate pathogens and keep long-term immunity [90]. Effector T cells are roughly divided into several subsets, including CD4+ helper T cells (Th1, Th2, Th17), CD8+ T cells and Tregs [91, 92]. Memory T cells are commonly classified into two categories: central memory T cells and effector memory T cells. Human naïve and memory T cells express CD45RA and CD45RO, respectively [93]. Several components of the non-canonical NF-κB pathway, like NIK, NF-κB2 and p52, have been confirmed to participate in T cell activation and T cell-mediated immunity [94–96]. Similarly, RelB-deficient mice presented with damaged T cell immunity, a reduction of interferon-γ (IFN-γ) and multiorgan inflammation [53, 62]. Emerging evidence reveals that RelB plays a negative role in Th17 differentiation [63]. Kurosawa et al. observed that effector memory cells from RelB-deficient mice displayed significantly elevated migratory activity than that in the WT mice [64]. Humans with RelB-deficiency present with T cells dysmaturity, reduced output of T cells from thymus and abnormal clonal expansion of T cell subtypes, which results in severe T cell immunodeficiency [48]. Specifically, complex phenotypes were observed, including increased memory cells, weakened T cell responses, significantly reduced IFN-γ and IL-2 generation and decreased expression of signal transducer and activator of transcription 1 (STAT1) and T-bet, which facilitate Th1 differentiation [48]. Taken together, RelB has a pleiotropic effect on T cell differentiation and T cell-mediated immunity.

#### B cell development, survival, germinal center formation and humoral immunity

B cells are essential for humoral immunity. After T cell-dependent antigenic stimulation, GC B cells undergo somatic hypermutation, negative selection and eventually differentiate into memory B cells and high-affinity plasma cells, which are responsible for immunological memory formation and antigen-specific antibody secretion, respectively [97, 98]. FDCs are limited to GCs and contribute to negative selection of B cells by expressing antigens on their surfaces [98]. In addition, marginal zone B cells play a critical role in T cell-independent humoral immune response [99]. GC and FDC network shortages, follicular B cell reduction and marginal zone B cell absence can be found in the spleens of RelB-deficient mice [56]. Consistently, mice with combined deficiency of RelB/NF-κB2 or RelB/cRel also show B-cell progenitor developmental disorders, the tumble of established GCs and remarkable reduction of peripheral mature B cells [65, 66], which were also observed in BAFF or BAFFR-deficient mice [100–104]. Weih et al. illustrated that RelB activation in stromal cells was responsible for the formation of GCs and FDC networks, whereas RelB expression in hemopoietic cells was required for the generation of marginal zone B cells [56]. RelB mediates GC B cell maturation via CD40 and BAFFR signaling [65, 67] and maintains B-cell survival via BAFFR signaling [65, 66]. BAFFR-mediated survival signaling in mature B cells functions by activating the non-canonical pathway [66, 105, 106]. In humans with RelB deficiency, B cell development is halted and CD27+ memory B cells are absent, leading to shortage of specific antibodies and severe B cell immunodeficiency [8, 48]. Furthermore, B cells from these patients show decreased expression of the surface molecule BAFFR and impaired CD40 signaling [48]. Collectively, RelB plays a crucial role in B cell development, survival, GC formation and humoral immunity.

## RelB in the pathogenesis of multiple sclerosis and its animal model

**Fig. 2:**
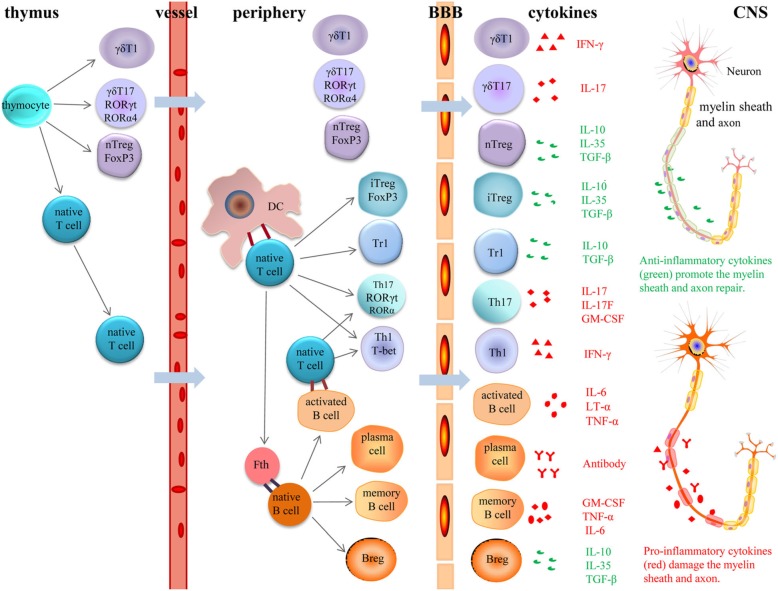
Role of different cells in the pathogenesis of MS and EAE. In the thymus, thymocyte precursor cells develop into γδT1, γδT17, nTregs and naïve CD4+ T cells. Upon neuroinflammation, γδT1 and γδT17 cells can cross the endothelial BBB and traffic into the central nervous system CNS, whereas naïve T cells migrate into the peripheral immune tissue. Naïve T cells connected with APCs (DCs and B cells), thereby differentiating into various effector T cells (iTregs, Tr1, Th17 and Th1). Th1, Th17, γδT1 and γδT17 cells secrete pro-inflammatory cytokines that trigger neuroinflammation and impair the myelin sheath and axons. Meanwhile, Tregs (Tr1, iTregs and nTregs) secrete anti-inflammatory cytokines and restrain immune responses mediated by T cells, B cells and DCs, thereby promoting tissue repair. Further, with the help of Tfh cells, naïve B cells differentiate into plasma cells, memory B cells and Bregs. While plasma cells damage the myelin sheath and axons on neurons via secreting antibodies, Bregs play a protective role via producing IL10, IL35 and TGF-β. Memory B cells and several activated B cells can produce a series of pathogenic cytokines Abbreviations: MS: multiple sclerosis; EAE: experimental autoimmune encephalomyelitis; γδ: gamma delta; BBB: blood-brain barrier; CNS: central nervous system; APC: professional antigen presenting cells; DC: dendritic cell; iTreg: induced regulatory T cell; Tr1: type 1 Treg; Th: T helper; nTreg: natural regulatory T cell; Tfh: follicular helper T; Breg: regulatory B cell; IL: interleukin; IFN-γ: interferon-γ; TGF-β: transforming growth factor-β; TNF-α: tumor necrosis factor α; GM-CSF: granulocyte monocyte-colony stimulating factor

MS is a chronic and progressive autoimmune disease in the CNS, characterized by inflammatory demyelination of the brain and spinal cord [10]. Experimental autoimmune encephalomyelitis (EAE), a conventional animal model of MS, is widely applied to study the pathophysiology and treatment for MS [107]. MS is traditionally thought of as a T cell-mediated autoimmune disorder [108]. Autologous myelin antigen-derived CD4+ T cells migrate from the periphery into the CNS, where they produce cytokines, chemokines and inflammatory molecules to impair the myelin sheath and axons [109]. It is generally accepted that Th1 and Th17 cells are responsible for MS and EAE initiation [109, 110]. However, a growing body of evidence suggests that the occurrence of MS is always accompanied by diverse immune cells infiltration, which mainly contains a variety of activated T cells subtypes, DCs and B cells (Fig. 2) [10]. The invading immune cells mediate MS pathology by secreting a mass of pro-inflammatory or anti-inflammatory cytokines (Fig. 2) [111]. RelB is a powerful molecule that regulates lymphoid organ formation, as well as lymphocyte development and function [9]. While the relationship between RelB and a variety of tumors is widely studied, such as in laryngeal cancer, lung adenocarcinoma and colon cancer [112–115], the role of RelB in MS is still obscure. This section summarizes the RelB-associated mechanism in MS and EAE, which may provide new insights into the treatment of MS.

### Th17 cells

Th17 cells play a central role in the pathogenesis of MS, and their differentiation depends on the transactivation of the orphan nuclear receptors γt and α (RORγt and RORα) [116, 117]. Autoreactive Th17 cells infiltrate the CNS, where they secrete IL-17A, IL-17F and granulocyte monocyte-colony stimulating factor (GM-CSF). They attract and activate diverse immune cells, eventually resulting in neuroinflammation [118–121]. IL-17A (also known as IL-17), a hallmark cytokine of Th17 cells, is crucial for the development of EAE [122]. Functional blockage of IL-17 leads to a remissive disease course and improves the outcomes of EAE [123–125], whereas its high expression is related to MS severity [126]. GM-CSF^−/−^ mice fail to induce EAE because of the inability of autoreactive lymphocytes to proliferate and the ceasing of immune cell infiltration [120, 127]. The expression of GM-CSF and its receptor is upregulated in brain tissues from acute and chronic MS patients [128]. Interestingly, a recent study puts forwards a new theory that Th1-like Th17 effector memory cells, especially Th17.1 cells, dominantly contribute to MS pathology. Th17.1 cells were affluent and showed increased production of IFN-γ and GM-CSF in patients with MS [129].

There is growing evidence that RelB is a negative factor regarding mediating Th17 cell differentiation and function, which affects the induction and progression of EAE [63, 130, 131]. Park et al. showed that lipopolysaccharides directly stimulated Th17 cell differentiation, enhanced the frequency of IL-17-producing cells and aggravated EAE via modulating phosphorylation of RelB and NF-κB1 [132]. Xiao and his colleagues reported that OX40, a T cell costimulatory molecule in the TNFSFR family, activated downstream molecule RelB, which inhibited IL-17 expression and alleviated EAE through triggering chromatin modification and forming a “closed” chromatin structure at the IL-17 gene [131]. Besides, mucosa-associated lymphoid tissue lymphoma translocation gene 1 (MALT1) is closely related to the level of RelB protein. Using MALT1^−/−^ Th cells and mice, Brustle et al. demonstrated that the deficiency of MALT1 can reduce the degradation of RelB in Th17 cells and decrease the production of IL17 and GM-CSF, thereby preventing mice from EAE induction [130]. In conclusion, RelB regulates Th17 differentiation negatively in EAE, and the treatment that activates RelB in Th17 cells may be a potentially therapy for MS.

### Th1 cells

Prior to the discovery of Th17 cells, Th1 cells were thought to be responsible for neuroinflammation in MS and EAE [133]. Th1 cells express the transcription factor T-bet that acts as a positive regulator to promote IFN-γ production [134]. The level of IFN-γ could be increased in EAE and MS patients [135, 136]. Accumulating evidence indicates that both IFN-γ inactivation [137–140] and T-bet depletion [141] protect mice from developing EAE. Furthermore, Cron and his colleagues observed a noteworthy reduction of IFN-γ and defective Th1 differentiation in RelB-deficient mice. Meanwhile, a remarkable reduction in expression of T-bet occurs in RelB-deficient Th1 cells [142]. Collectively, RelB plays a role in MS and EAE by mediating T-bet expression and Th1 differentiation. Exploring approaches to suppress RelB expression in Th1 cells may be an implicit treatment for MS.

### Gamma delta T cells

In the thymus, some of the double negative thymocytes differentiate into gamma delta (γδ) T cells that further develop into IFN-γ-secreting (γδT1) and IL-17-secreting (γδT17) cells under the conditions of the transactivation of T-bet and the orphan nuclear receptors RORγt and RORα4, respectively [143, 144]. γδ T cells play an important role in the pathogenesis of MS and EAE [145, 146]. By using frozen CNS specimens from acute MS patients, Wucherpfennig et al. observed the accumulation and clonal expansion of γδ T cells in lesions [147]. Coincidentally, recent investigations indicated that γδ T cells exert a harmful effect on EAE, evidenced by a significant remission of the clinical symptoms in γδ T cell-deficient animals [148–150]. Some evidence demonstrated that γδT17 cells aggravate EAE by enhancing IL-17 production, suppressing Tregs responses and improving antigen-specific T cell responses [151]. Interestingly, another study has illustrated that RelB regulates γδT17 cell differentiation in the thymus and IL-17 production through controlling the expression of RORγt and RORα4, which requires the activation of LTβR signaling [144]. Therefore, RelB may worsen EAE by regulating γδT17 cell differentiation.

### Regulatory T cells and steady-state migratory DCs

Tregs are well accepted for their central role in restraining autoreactive immune responses and maintaining peripheral tolerance. There are two widely-studied types of Tregs: FoxP3+ Tregs and IL-10-secreating type 1 Tregs (Tr1) [152]. FoxP3+ Tregs encompass two categories: thymus-derived natural Tregs (nTregs) and periphery-induced Tregs (iTregs), and both of them secrete immunosuppressive cytokines, such as IL-10, IL-35 and transforming growth factor-β (TGF-β) [153]. Tr1 cells are induced in the periphery and primarily produce IL-10 and TGF-β [154]. Tregs can restrain various immune responses mediated by T cells, B cells and DCs [155]. The protective role of FoxP3+ Tregs and Tr1 cells was observed in MS and EAE [156–160].

Mature DCs are essential for the immune response, whereas immature DCs improve immune tolerance by inducing T cell anergy or Tregs generation [161]. Steady-state migratory DCs, known as semi-mature DCs, transport self-antigens from peripheral tissues to the draining lymph nodes [162]. Idoyaga et al. proved that steady state Langerin+ migratory skin DCs exert an unparalleled effect on inducing Foxp3+ Treg generation in vivo, which prominently improved the prognosis of EAE [163, 164]. Moreover, the activation of the non-canonical NF-κB pathway via RelB/p52 is essential for maintaining the frequency of steady-state migratory DCs and inducing Foxp3+ iTreg formation by steady state migratory RelB+ Langerin+ dermal DCs [165]. RelB+ Langerin− dermal DC subset controls the peripheral pool of Foxp3+ nTregs [166]. Further, some researchers found Foxp3+ Tregs markedly expanded in mice with RelB depletion because of increased levels of IL-2, a growth factor for Foxp3+ Tregs that is produced by hyperactive T effector cells [167]. In conclusions, the role of RelB in EAE via regulated Tregs and steady-state migratory DCs are complex. Further studies are still needed to uncover exact mechanisms, and steady-state migratory DCs may be a therapeutic target for MS or other autoimmune diseases.

### Dendritic cells

DCs play a critical role in activating immune response. In MS and EAE, DCs present autologous myelin antigen to naïve CD4+ T cells, which then differentiate into myelin-reactive Th1 and Th17 cells that induce neuroinflammation and CNS damage [168–170]. Moreover, one of their major effector molecules, cytokine IL-23, is also increased in MS patients [171]. RelB is essential for DC maturation [86], and silencing RelB generates stable tolerogenic properties, creating what is known as tolerogenic DCs [172]. Tolerogenic DCs exhibit an immature phenotype, with lower levels of costimulatory molecules, repressed effector T cell responses and Treg induction [172]. In a previous study, our lab successfully induced tolerogenic DCs by applying 1,25-(OH)_2_D_3_, which repressed EAE via the induction of Tregs and the reduction of Th1/Th17 [173]. Coincidentally, monocyte-derived DCs that were treated with 1,25-(OH)_2_D_3_ also showed a decreased ability to induce a T cell response and an increase in anti-inflammatory cytokines in MS patients, compared to healthy controls [174]. Moreover, 1,25-(OH)_2_D_3_ may function by regulating RelB expression in DCs [23, 175]. In addition, dimethyl fumarate (DMF), an immunotherapeutic drug for MS approved by the United States Food and Drug Administration (FDA), has a therapeutic effect via impairing human myeloid DC maturation. Compared to untreated cells, myeloid DCs from DMF-treated MS patients showed an immature phenotype, decreased expression of RelB, limited capacity to activate T cells and reduced secretion of pro-inflammatory cytokines IFN-γ, IL-17 and GM-CSF [176]. Currently, RelB-silenced tolerogenic DCs are used to study autoimmune diseases, such as systemic lupus erythematosus and myasthenia gravis. Moreover, significant protective effects have been observed in disease conditions [177–179]. In a very recent research, Andreas and his team found that mice with RelB-deficient DCs are almost resistant to induction of the EAE model because of the accumulation of FoxP3+ Tregs and the reduction of pathogenic T cells [180]. Therefore, RelB-silenced tolerogenic DCs may be a promising cell therapy for MS.

It should be noted that macrophages are also effective antigen-presenting cells and Recent reports indicate that macrophages play dual roles in the pathogenesis of MS as they contribute to lesion formation and axonal damage, but also present repair mechanisms through the production of neurotrophic factors and anti-inflammatory molecules as well as clearance of myelin debris (Abdul-Majid et al.,2002; Kigerl et al., 2009).

### B cells

For decades, MS was generally known as a mainly T cell-mediated disease, and the role of B cells in MS was overlooked; however, a growing amount of evidence shows the significant involvement of B cells in MS pathology [181, 182]. When naïve B cells encounter myelin antigens, they are activated and differentiate into plasma cells, memory B cells and regulatory B cells (Bregs) with the help of follicular helper T (Tfh) cells [108]. In MS patients, a portion of activated B cells act as APCs, presenting myelin antigens to CD4+ T cells and improving Th1 and Th17 responses [183–185]. Plasma cells produce myelin specific antibodies that not only cause functional myelin impairment, but also form oligoclonal bands (OCBs) within the CNS and peripheral blood [186]. The detection of OCBs from cerebrospinal fluid are used to diagnose MS with high sensitivity [187, 188]. In untreated RRMS patients, circulating effector memory B cells significantly increase, producing abundant GM-CSF, TNF-α and IL-6 [189]. Additionally, activated B cells from MS patients or EAE mice also secrete pathogenic cytokines IL-6, TNF-α and LT-α [189, 190]. By contrast, Bregs play a protective role in EAE by producing several anti-inflammatory cytokines, such as IL-10, IL-35 and TGF-β [191–194]. RelB is critical for the maturation and survival of B cells. Mice and humans with RelB deficiency present with developmental disorders of B-cell progenitors and a significant reduction of peripheral mature B cells [48, 65, 66]. Furthermore, RelB/NF-κB2-deficient GC B cells have reduced the expression of inducible T cell co-stimulator ligand (ICOSL), which connect with ICOS expressed on Tfh cells to mediate the selection of high-affinity B cells [195]. Hence, the inhibition of RelB expression in B cells may be beneficial for MS.

### Other immune cells

Macrophages and microglia are prominent innate immune cells and play a dual role in the pathogenesis of MS [196]. While macrophages and microglia are induced into the M1 phenotype in the acute phase, which contribute to demyelination and MS lesion formation; macrophages and microglia are activated into M2 phenotype in the later stage, which exhibit neuroprotective effect by clearance of myelin debris and secretion of neuroprotective molecules [196, 197]. Therefore, shifting the phenotype of macrophages and microglia from M1 into M2 may be attractive therapy for MS. Interestingly, overexpression of RelB was observed in LPS-stimulated macrophages, which suppressed the production of TNFα, a pro-inflammatory cytokine [198]. Some studies also observed that the expression of RelB was enhanced in M1 macrophages and RelB deficiency inhibited the differentiation of M1 macrophage [199, 200]. Therefore, strategies to degrade RelB could suppress the polarization of macrophages toward pro-inflammatory phenotype M1 cells, which might be beneficial for the trentment of MS.

### Non-immune CNS cells

The role of oligodendrocytes and astrocytes in MS cannot be ignored by researchers. MS lesions are featured by oligodendrocyte death and axon degeneration. Gupta et al. found that the deficiency of RelB in oligodendrocytes decreased the severity of EAE through promoting survival of mature oligodendrocytes [201]. As the most abundant cell type in the CNS, astrocytes are important regulators of inflammation and essential for maintaining CNS homeostasis [202]. Highly expressed RelB in astrocytes may induce immune tolerance in experimental neuroinflammation due to decreased pro-inflammatory cytokines such as IL-1β, IL-6 and IL-8 [203]. Moreover, the severity of EAE with RelB specifically deleted in astrocytes is similar with control mice [201]. Taken together, regulating the expression of RelB in oligodendrocytes and astrocytes may be an option to treat MS in the future.

## RelB as a future therapeutic target for MS

As mentioned above, RelB has pleiotropic effects on MS or EAE pathogenesis via a cell type-specific manner. RelB activation or inhibition in specific cell types could be achieved by regulating upstream signaling pathway. While decreased RelB expression in Th1, γδT17, DCs, B cells, macrophages and oligodendrocytes may have a beneficial role in MS or the EAE animal model, suppressive processes in other cell types may also cause greater severity. In this scenario, the use of RelB inhibitors in vivo is still in its infancy, and potential harmful effects must not be ignored by researchers. Therefore, we propose that targeted therapies in more specific cell types, such as RelB-inhibited Th1, γδT17, DCs, B cells, macrophages and oligodendrocytes need to be further investigated. Considering the fact that our research group has successfully alleviated established EAE by adoptive transfer of 1,25-(OH)_2_D_3_-induced tolerogenic DCs [173], in the future, the adoptive transfer of RelB-silenced tolerogenic DCs may be a promising strategy for the precise treatment of MS. However, to our knowledge, limited data about RelB in patients with MS has been reported and most work has been mainly focused on the animal models of MS. Only when we are sufficiently knowledgeable should we consider targeting RelB as a clinical approach to treat patients with MS.

## Conclusion

Transcription factor RelB, a member of NF-κB family, is essential for lymphoid organ formation and lymphocyte development and function. In MS and its animal model EAE, RelB exerts an impact on Th17, Th1, γδT17, steady-state migratory DCs, DCs, B cells, macrophages, microglia, oligodendrocytes and astrocytes, which provide the theoretic foundation for possible therapies that target RelB. Further studies are still needed to better understand RelB-associated mechanisms and applications.

## Data Availability

All work cited is in the public domain.
